# Overfeeding‐induced weight gain elicits decreases in sex hormone‐binding globulin in healthy males—Implications for body fat distribution

**DOI:** 10.14814/phy2.15127

**Published:** 2021-12-08

**Authors:** Prachi Singh, Naima Covassin, Fatima H. Sert‐Kuniyoshi, Kara L. Marlatt, Abel Romero‐Corral, Diane E. Davison, Ravinder J. Singh, Michael D. Jensen, Virend K. Somers

**Affiliations:** ^1^ Department of Cardiovascular Medicine Mayo Clinic Rochester Minnesota USA; ^2^ Pennington Biomedical Research Center Louisiana State University System Baton Rouge Louisina USA; ^3^ Department of Laboratory Medicine and Pathology Mayo Clinic Rochester Minnesota USA; ^4^ Division of Endocrinology Mayo Clinic Rochester Minnesota USA

**Keywords:** estrogen, regional fat distribution, sex hormone‐binding globulin, testosterone, weight gain

## Abstract

**Objective:**

Obesity and upper‐body fat elevates cardiometabolic risk. However, mechanisms predisposing to upper‐body fat accumulation are not completely understood. In males, low testosterone (T) frequently associates with obesity, and estrogen deficiency may contribute to upper‐body adiposity. This study examines the effects of overfeeding‐induced weight gain on changes in gonadal hormones in healthy males and its association with regional fat depots.

**Methods:**

Twenty‐five males (age: 29.7 ± 6.9 years; BMI: 24.7 ± 3.1 kg/m^2^) were overfed for 8 weeks to gain approximately 5% body weight. Changes in total and regional fat depots were assessed using dual‐energy x‐ray absorptiometry and abdominal computed tomography scans. Circulating T, estrone (E1), 17‐β estradiol (E2), and sex hormone‐binding globulin (SHBG) concentrations were measured at baseline and after weight gain.

**Results:**

Overfeeding resulted in 3.8 (3.3, 4.9) kg weight gain with increased total body fat. Weight gain did not alter circulating T (*p* = 0.82), E1 (*p* = 0.52), or E2 (*p* = 0.28). However, SHBG decreased (*p* = 0.04) along with consequent increases in T/SHBG (*p* = 0.02) and E2/SHBG (*p* = 0.03) ratios. Importantly, baseline E2/SHBG ratio was inversely associated with increases in upper‐body fat mass (*ρ* = −0.43, *p* = 0.03).

**Conclusions:**

Modest weight gain does not alter circulating gonadal hormones in males but may increase bioavailability of T and E2 via decreases in SHBG. The association between baseline E2/SHBG and regional fat mass suggests that higher levels of bioavailable E2 may protect from upper‐body fat accumulation during overfeeding‐induced modest weight gain in healthy males. Our study suggests a complex relationship between adipose tissue, gonadal hormones, and fat accumulation in males.

## Graphical abstract

We show that modest overfeeding‐mediated weight gain does not alter circulating sex hormones but may increase bioavailable hormone via decreases in sex hormone‐binding globulin (SHBG). Notably, the decreases in SHBG may be partly mediated by changes in leptin. Lastly, we observed a negative relationship between bioavailable estradiol and increases in upper‐body fat during overfeeding which suggests that estradiol may protect from increases in upper‐body fat mass during overfeeding in males.

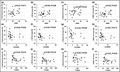

## 1 INTRODUCTION

Body fat distribution is an important predictor of cardiovascular risk (Bray et al., 2018; Chen et al., 2019; Despres, 2012; Fox et al., 2007; Liu et al., 2010). Specifically, upper‐body or abdominal fat is associated with increased cardiovascular risk and cardiovascular mortality across a range of body weight (Britton et al., 2013; Chen et al., 2019; Sahakyan et al., 2015; Sun et al., 2019). The adverse effect of abdominal fat on cardiovascular risk is also supported by experimental overfeeding studies, which show that increases in visceral fat following modest weight gain are associated with vascular dysfunction and blood pressure elevation in healthy individuals (Covassin et al., 2018; Romero‐Corral et al., 2010). However, mechanisms related to gains in upper‐body subcutaneous and visceral fat are not completely understood.

Gonadal hormones, particularly testosterone (T), are associated with obesity and cardiovascular risk. Low T is more common in obesity and is considered an independent risk factor for cardiovascular events, cardiovascular mortality, and all‐cause mortality (Eriksson et al., 2017; Haring et al., 2010; Khaw et al., 2007; Laughlin et al., 2008). The ability of weight loss to reverse obesity‐associated hypogonadism indicates that the inverse relationship between T and obesity is likely due to obesity (Corona et al., 2013). Hypogonadal males also have reduced fat‐free mass and increased abdominal fat and total body fat, which are improved by T replacement (Corona et al., 2016; Kelly & Jones, 2015). Notably, experimental studies examining the effects of modest weight gain on circulating gonadal hormones are lacking. These would be important to determine causality and identify strategies which may improve cardiometabolic profile.

Hypogonadal males frequently have both T and estrogen deficiency (Beld et al., 2000; Khosla et al., 2001). These decreases in estrogen are partly related to the decreased aromatization possible with lower T availability. In males, T largely contributes to circulating estrogens (Longcope et al., 1969). Therefore, it is likely that the impact of low T on body composition may be mediated by decreases in estrogen. Indeed, experimental studies examining the differential role of estrogen versus T deficiency show that decreases in estrogens primarily account for increases in fat mass in healthy males (Chao et al., 2016; Finkelstein et al., 2013). Together, these findings provide a strong rationale to examine the effects of weight gain on T and estrogens in healthy males. The overall objective of the study is to determine the relationship between gonadal hormones and regional body fat distribution during conditions of energy excess. In this study, we report the changes in T, estrone (E1), and 17‐β estradiol (E2) in response to experimental overfeeding‐induced weight gain. Because sex hormone‐binding globulin (SHBG) determines the bioavailability of these hormones, we also measured SHBG. We hypothesized that weight gain would elicit decreases in T, E1, and E2 along with decreases in SHBG.

## 2 MATERIALS AND METHODS

### 2.1 Subjects

Healthy males (*n* = 25, age: 29.7 ± 6.9 years, range 20–50 years; BMI: 24.7 ± 3.1 kg/m^2^, range 18.9–34.3 kg/m^2^) were recruited from the Rochester community as part of a larger study examining the cardiovascular disease mechanisms associated with weight gain (NCT00589498). To be eligible, participants needed to be sedentary, non‐smoking, and free of chronic diseases. Exclusionary criteria included: (a) known sleep disorders; (b) chronic use of any prescription medications; and (c) shift work. The research protocol was approved by the Mayo Clinic Institutional Review Board and written informed consent was obtained from all participants. All study procedures were conducted at the Clinical Research and Trials Unit at Mayo Clinic Rochester in accordance with the ethical standards on human experimentation between 2004 and 2010. Findings from the larger study relating to endothelial function, blood pressure, heart rate variability, and adipose tissue cellularity have been published (Adachi et al., 2011; Covassin et al., 2018; Romero‐Corral et al., 2010; Singh et al., 2012). The present manuscript examining the changes in gonadal hormones with experimental weight gain includes all male participants who were randomized to the overfeeding intervention. Gonadal hormones from females participating in this study were not measured because the study did not time blood sample collections for specific times of the menstrual cycle.

### 2.2 Study design

All participants underwent physical examinations after medical history was obtained to confirm eligibility. Screening procedures also included overnight polysomnography to confirm absence of sleep disorders. Prior to baseline assessments, participants met with a research dietician to establish a formal eating schedule that they would follow for 3 consecutive days to achieve energy balance. Energy requirements were determined by registered dieticians using the Harris–Benedict equation plus occupational activity. All meals were prepared by the research kitchen and were of the same macronutrient composition (40% carbohydrate, 40% fat, and 20% protein) (Singh et al., 2012). After the baseline period, participants underwent 8 weeks of overfeeding to gain weight. The goal of the overfeeding intervention was to increase total body weight by ~5% over a period of 8 weeks. During this period, participants were asked to maintain their usual physical activity and consume additional calories through 1–3 dietary add‐ons to their usual daily food intake. The add‐ons were provided from the research kitchen and included a choice of an ice cream shake (402 kcal), a king‐size chocolate bar (510 kcal), and a nutritional energy drink (360 kcal/8 oz). Body weight was measured at least five times per week at the Clinical Research and Trials Unit to ensure gradual weight gain and to inform whether adjustments to the consumption of the number of supplements per day were needed to achieve the targeted increases in body weight. Body composition and blood measurements were obtained at baseline and after 8 weeks of weight gain.

### 2.3 Body composition

Total body composition was determined using dual‐energy x‐ray absorptiometry (DXA, DPX‐IQ, Lunar Radiation). Duplicate scans were obtained at baseline and after 8 weeks of overfeeding. Areas defining lower‐body and abdomen were manually set for each scan and average values for each study time point were used for analysis. Upper‐body fat mass was calculated by subtracting the lower‐body fat mass from total body fat mass. Measurement of total abdominal and visceral fat area was obtained using average values determined from computed tomography (CT) at the levels of L_2–3,_ L_3–4_, and L_4–5_. Visceral fat mass was estimated by combining data from DXA and CT as previously described (Singh et al., 2012).

### 2.4 Blood measurements

Fasting morning blood samples were used to measure T, E1, E2, and SHBG at the Mayo Clinic Immunochemical core laboratory. High‐sensitivity T was measured by liquid chromatography–tandem mass spectrometry (LC–MS/MS) (Agilent Technologies). For estrogens, E1 and E2 were extracted with methylene chloride and after derivatization with dansyl chloride. High‐pressure LC was used prior to introduction of the derivatized sample extract into the tandem mass spectrometry (LC–MS/MS) (Agilent Technologies). SHBG was measured by a solid phase, two‐site chemiluminescent assay on the Siemens Immulite 2000 automated immunoassay system (Siemens Healthcare Diagnostics). Measurement of lipid profile, leptin, insulin, and glucose was undertaken using standard methods as previously described (Romero‐Corral et al., 2010).

### 2.5 Statistical analysis

Data are summarized as median and interquartile range. Variables measured at baseline were compared to weight gain values using the paired Wilcoxon signed‐rank test. The relationships between sex hormones and regional fat depots were evaluated using the Spearman's correlation coefficient. Nonparametric tests were used to assess changes in measures because of the limited sample size and unmet assumptions including normal distribution. JMP Pro 14.2.0 (SAS Institute, Inc.) was used for statistical analysis. Primary outcomes examined the effects of weight gain on sex hormones (circulating and bioavailable) and SHBG. Secondary outcomes evaluated (a) the associations between sex hormones, SHBG, and regional fat depots and (b) the relationship between changes in sex hormones and SHBG with leptin and insulin. Considering the exploratory nature of the study, significance level was set a priori at *p* < 0.05.

## 3 RESULTS

Participants gained 3.8 (3.3, 4.9) kg body weight with overfeeding, which resulted from increases in total fat mass; increases were seen in all regional fat depots. Fat‐free mass remained unchanged (Table 1). Among the circulating metabolic variables measured, only leptin increased significantly with weight gain.

**TABLE 1:** Effects of overfeeding in study participants

Variable	Baseline	Weight gain	*p*
Weight (kg)	78.1 (72.6, 84.1)	84.2 (76.5, 88.7)	<0.0001
BMI (kg/m^2^)	24.5 (23.9, 25.6)	25.8 (25.0, 26.7)	<0.0001
Percent body fat (%)	20.4 (16.7, 31.9)	23.5 (19.9, 35.8)	<0.0001
Total body fat mass (kg)	15.4 (12.2, 25.4)	20.6 (15.7, 28.2)	<0.0001
Total body fat‐free mass (kg)	57.9 (50.4, 66.3)	58.9 (51.4, 67.6)	0.15
LB fat mass (kg)	6.7 (5.2, 10.6)	7.4 (6.1, 11.3)	<0.0001
Visceral fat mass (kg)	1.7 (1.0, 2.8)	2.1 (1.4, 3.5)	0.002
UBSC fat mass (kg)	7.1 (5.6, 12.5)	9.7 (7.1, 13.5)	<0.0001
Total cholesterol (mg/dl)	164 (127, 182)	171 (137, 189)	0.18
High‐density lipoprotein (mg/dl)	38 (34, 43)	38 (28, 45)	0.66
Triglycerides (mg/dl)	81 (74, 107)	77 (64, 141)	0.67
Low‐density lipoprotein (mg/dl)	103 (84, 116)	102 (86, 130)	0.16
Glucose (mg/dl)^a^	92 (90, 97)	96 (93, 102)	0.17
Insulin (μU/ml)^a^	4.6 (3.3, 7.0)	5.4 (3.7, 9.3)	0.18
Leptin (ng/ml)^a^	3.1 (1.7, 6.5)	4.8 (3.5, 8.7)	0.0001
T (nmol/L)	18.01 (14.70, 23.89)	17.07 (14.80, 22.43)	0.82
E1 (pmol/L)	103.54 (85.05, 140.5)	118.34 (92.45, 142.37)	0.52
E2 (pmol/L)	88.10 (71.59, 100.95)	95.45 (73.42, 113.80)	0.28
SHBG (nmol/L)	20.50 (14.65, 33.05)	17.30 (12.10, 30.30)	0.04

Note

All values are median (interquartile range). *p* is calculated using paired the Wilcoxon signed‐rank test. *N* = 25.

Abbreviations: E1, estrone; E2, 17‐β estradiol; LB, lower‐body; SHBG, sex hormone‐binding globulin; T, testosterone; UBSC, upper‐body subcutaneous.

^a^ From *N* = 24.

### 3.1 Weight gain and sex hormones

**FIGURE 1:**
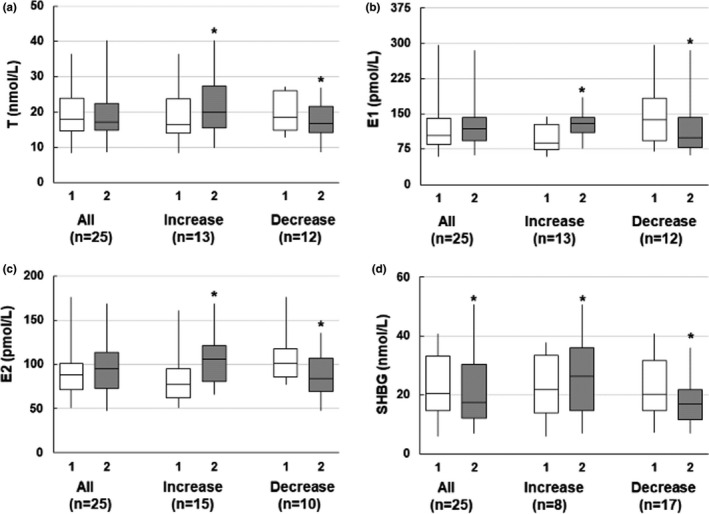
Interindividual variations in sex hormones in response to overfeeding‐induced weight gain in healthy men (*n* = 25). Overall, testosterone (T, a), estrone (E1, b), and 17‐β estradiol (E2, c) did not change with weight gain. However, compared to baseline pre‐interventional values, decreases in T and E1 were evident in 12 participants while increases in E2 were observed in 15 individuals. Furthermore, decrease in sex hormone‐binding globulin (SHBG, d) with weight gain was observed. Data are presented as median and interquartile range. Fiskars depict minimum and maximum values. 1: Baseline, White bars; 2: post‐8‐week overfeeding, gray bars. *p* values were calculated using the paired Wilcoxon signed‐rank test. * is *p* < 0.05. Groups depicting increase or decrease for each variable are presented to highlight the variability in response to overfeeding

T and estrogens (E1 and E2) did not change in response to overfeeding‐induced weight gain (Table 1). However, marked interindividual variability in response to excessive calorie intake was observed (Figure 1). Of the 25 participants, an increase in T was seen in 13 individuals (ΔT 12.4 [4.3, 19.9]%), and a decrease in T was seen in 12 individuals (ΔT: −9.4 [−19.4, −2.8]%) (Figure 1a). To gain insight into individual predictors of T response to weight gain, we examined the relationship between changes in T with measures of baseline body composition. Of note, relative changes in T during weight gain did not correlate with any baseline measure of body composition measured in the study (all *p* > 0.14). Similarly, interindividual variability in response to overfeeding was observed for E1 and E2. Increases in E1 were seen in 13 participants (ΔE1: 28.2 [8.7, 53.5]%), and no change or decrease in E1 was seen in 12 participants (ΔE1: −12.6 [−31.2, −0.9]%). Overfeeding‐induced weight gain caused increases in E2 in 15 participants (ΔE2: 26.9 [11.1, 55.0]%), and no changes or decreases were seen in 10 participants (ΔE2: −19.8 [−27.6, −4.5]%) (Figure 1b,c). Of note, consistent increase or decrease in T, E1, or E2 in response to weight gain was not observed in individuals. An inverse association between baseline estrogens and overfeeding‐induced changes in E1 (baseline E1: *ρ* = −0.62, *p* = 0.001; baseline E2: *ρ* = −0.55, *p* = 0.004) and E2 (baseline E1: *ρ* = −0.41, *p* = 0.04; baseline E2: *ρ* = −0.57, *p* = 0.003) was observed. Overfeeding‐induced changes in E1 and E2 did not correlate with any baseline measure of body composition (all *p* > 0.07). No association between relative changes in T, E1, and E2 with changes in leptin or insulin was observed.

Importantly, decreases in SHBG (−13.3 [−23.4, 4.7]%, *p* = 0.04, Table 1) and increases in E2/SHBG ratio (baseline vs. weight gain, 4.0 [3.0, 5.4] vs. 5.5 [3.1, 7.5], respectively, *p* = 0.03) and T/SHBG ratio (0.9 [0.7, 1.1] vs. 1.0 [0.8, 1.24], *p* = 0.02) were observed. With respect to interindividual variability, decreases in SHBG were seen in 17 participants (ΔSHBG: −19.2 [−24.9, −12.6]%), and increases were seen in 8 participants (ΔSHBG: 11.7 [4.2, 29.9]%, Figure 1d). Relative changes in SHBG did not correlate with any baseline measures of regional fat depots (all *p* > 0.70). Furthermore, while relative changes in SHBG showed a tendency to correlate with changes in T (*ρ* = 0.35; *p* = 0.09), no relationship was observed for E1 (*ρ* = 0.17, *p* = 0.43) and E2 (*ρ* = 0.26, *p* = 0.21).

**FIGURE 2:**
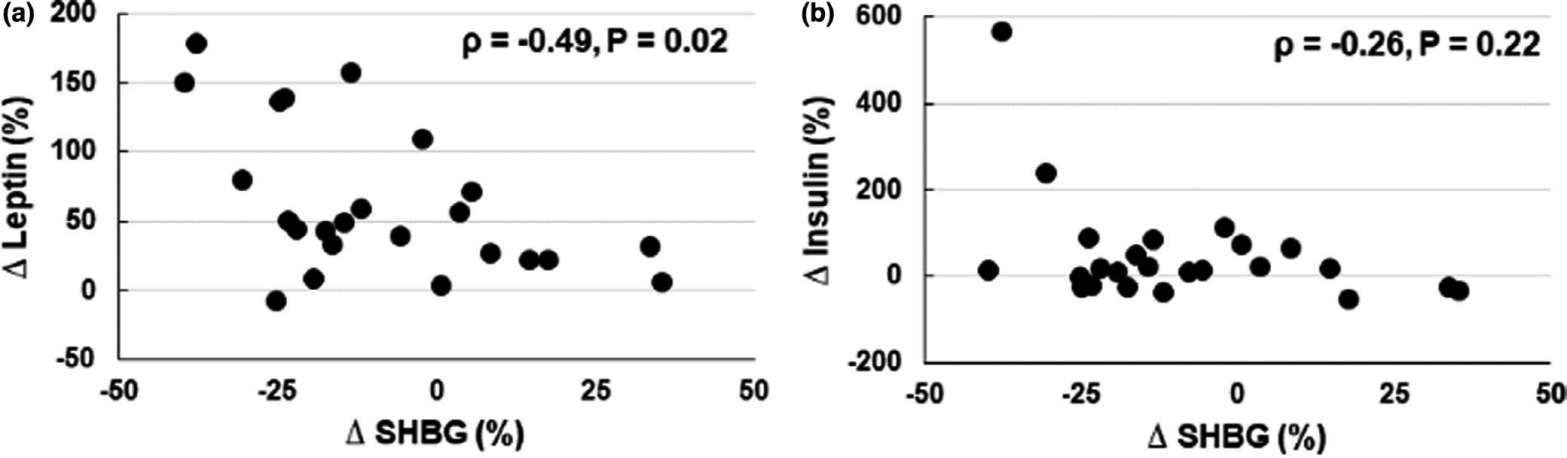
Relation between relative changes in leptin (a) and insulin (b) with relative changes in sex hormone‐binding globulin (SHBG) during weight gain. The relation was significant with changes with leptin but not significant with changes with insulin as determined using Spearman's correlation analysis. *N* = 24

To test for associations that might give insights into mechanisms for the changes in SHBG, we examined the relationship between changes in SHBG with changes in leptin and insulin. An inverse relationship between relative changes in leptin and SHBG was observed with weight gain (*ρ* = −0.49, *p* = 0.02) (Figure 2a). Relative changes in insulin did not correlate with changes in SHBG during weight gain (*ρ* = −0.26, *p* = 0.22, Figure 2b).

### 3.2 Relationships between sex hormones and regional fat depots

To gain understanding of the cross‐sectional and dynamic relationship between sex hormones and regional fat depots, we examined the individual associations of hormones with regional fat depots at baseline and relative changes with weight gain (Table 2). At baseline, T was inversely correlated with upper‐body fat mass (*ρ* = −0.42, *p* = 0.04) and visceral fat mass (*ρ* = −0.50, *p* = 0.01). These relationships were maintained after weight gain (upper‐body fat mass: *ρ* = −0.65, *p* = 0.001; visceral fat mass: *ρ* = −0.52, *p* = 0.007). However, relative changes in T during weight gain did not correlate with relative changes in any regional fat depot. E1 and E2 were not correlated with regional fat depots at baseline or changes with weight gain. An inverse relationship between SHBG and all regional fat depots was noted at baseline and after weight gain (all *p* < 0.05), but dynamic associations were not observed (all *p* > 0.16).

**TABLE 2:** Relationships between sex hormones, sex hormone‐binding globulin, and regional fat depots

	LB fat (kg)		UB fat (kg)		Visceral fat (kg)		UBSC fat (kg)	
	*ρ*	*p*	*ρ*	*p*	*ρ*	*p*	*ρ*	*p*
Baseline values								
T	−0.32	0.12	**−0.42**	**0.04**	**−0.50**	**0.01**	−0.35	0.08
E1	−0.05	0.80	−0.05	0.80	−0.20	0.34	0.01	0.97
E2	−0.15	0.46	−0.17	0.42	−0.29	0.15	−0.15	0.47
SHBG	**−0.60**	**0.00**	**−0.74**	**0.00**	**−0.67**	**0.00**	**−0.68**	**0.00**
Relative changes with weight gain								
T	0.11	0.61	0.06	0.79	0.14	0.52	−0.08	0.72
E1	−0.13	0.53	0.15	0.46	0.27	0.20	0.06	0.78
E2	−0.11	0.61	0.18	0.40	0.24	0.25	0.08	0.71
SHBG	−0.11	0.59	−0.18	0.39	0.10	0.63	−0.28	0.17

Note

*p* values were calculated using Spearman's correlation analysis. Significant correlations are bolded. *N* = 25.

Abbreviations: E1, estrone; E2, 17‐β estradiol; LB, lower‐body; SHBG, sex hormone‐binding globulin; T, testosterone; UB, upper‐body; UBSC, upper‐body subcutaneous.

**FIGURE 3:**
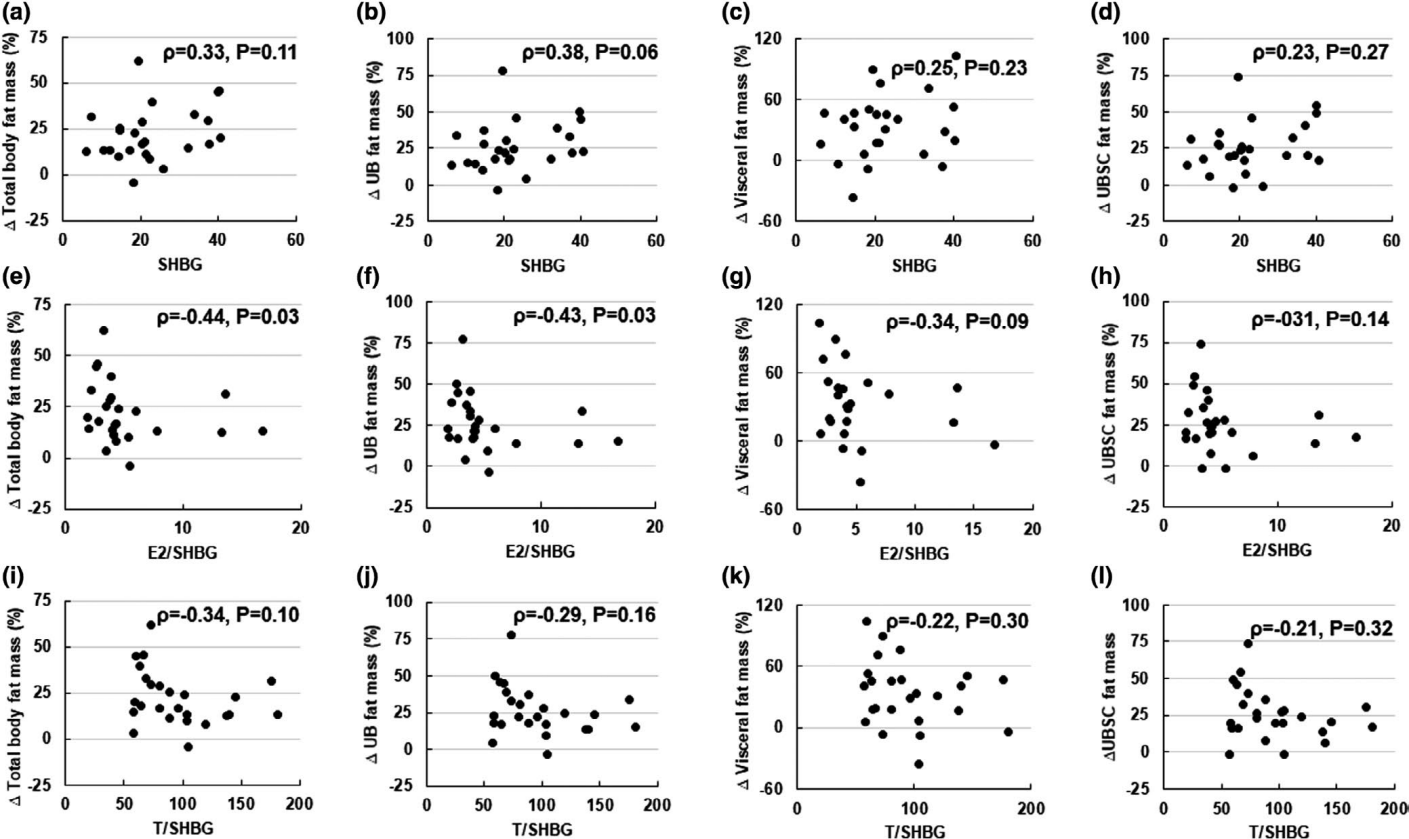
Associations between baseline sex hormone‐binding globulin (SHBG), bioavailable sex hormones, and relative changes in regional fat during weight gain. Baseline SHBG does not associate with changes in total body fat mass (a), visceral fat mass (c), and upper‐body subcutaneous (UBSC) fat mass (d). However, a tendency to positively associate with upper‐body (UB) fat mass was observed (b). Changes in total body fat mass (e) and UB fat mass (f) correlate with bioavailable 17‐β estradiol (E2/SHBG ratio) while changes in visceral fat mass (g) show a tendency to associate with E2/SHBG ratio. Changes in UBSC fat mass (h) does not correlate with E2/SHBG ratio. Bioavailable testosterone (T/SHBG ratio) at baseline does not correlate with changes in total body fat mass (i), UB fat mass (j), visceral fat mass (k), and UBSC fat mass (l). Correlations were determined using Spearman's correlation analysis. *N* = 25

Considering the changes in SHBG and consequent changes in bioavailability of estrogens and T with weight gain, we also examined the relationships between changes in E2/SHBG and T/SHBG with changes in regional fat depots. Increases in E2/SHBG ratio and T/SHBG ratio during weight gain did not correlate with increases in any fat depot (all *p* > 0.08). Interestingly, E2/SHBG ratio at baseline was negatively associated with increases in upper‐body fat mass (*ρ* = −0.43, *p* = 0.03, Figure 3) and showed a tendency to inversely associate with increases in visceral fat mass (*ρ* = −0.34, *p* = 0.09). Baseline values of T/SHBG ratio did not correlate with changes in any fat depot. While baseline SHBG did not correlate with total body fat mass, a tendency to positively associate with upper‐body fat mass was observed (*ρ* = 0.38, *p* = 0.06).

## 4 DISCUSSION

Modest weight gain as a result of overfeeding is not associated with any consistent changes in circulating T, E1, and E2 in healthy males. However, weight gain is accompanied by marked lowering of SHBG and, hence, likely increases in bioavailable T and E2 as determined by T/SHBG and E2/SHBG ratios.

Evidence supporting inverse relationships between T and obesity is derived from several cross‐sectional studies, as well as longitudinal studies of weight loss and T supplementation (Beld et al., 2000; Corona et al., 2013; Eriksson et al., 2017; Escobar‐Morreale et al., 2017; MacDonald et al., 2010; Wu et al., 2008). In our study, we noted a similar inverse relationship of circulating T with regional body fat mass at baseline and after weight gain. However, the dynamic association between increases in regional fat mass with changes in T was not apparent. In contrast to the cross‐sectional studies and contradictory to our hypothesis, we show that weight gain does not lower circulating T in healthy males. This may be related to our study design that aimed for a modest increase in body weight over a short 8‐week period. Indeed, previous studies suggest that alterations in circulating T emerge only following greater increases in BMI (Eriksson et al., 2017). Notably, we observed a large interindividual variability in response to weight gain in our study participants. However, participants who increased versus those who decreased T during overfeeding were similar in all aspects including body composition and circulating metabolic markers such as leptin and insulin at baseline. While hormones involved in the hypothalamic–pituitary–gonadal axis, including gonadotropins follicle‐stimulating hormone and luteinizing hormone, may predict changes in T during weight gain, these hormones were not measured.

Furthermore, considering that overall circulating T did not change with weight gain, it is not surprising that circulating E1 and E2 also did not change with weight gain in our study participants. We show an elevated interindividual variability of estrogens in response to weight gain with the majority of participants (60%) showing increases in estrogens. The increases in estrogens with increasing fat mass are supported by cross‐sectional studies showing a positive association between E2 and BMI along with reduction in E2 with weight loss (Bjornerem et al., 2004; Hammoud et al., 2009; Muller et al., 2003). The lack of increase in estrogens with weight gain among the remaining 40% of the study subjects may be partly related to baseline BMI. Participants with lower or no change of E2 with weight gain had a higher baseline BMI (26.2 ± 3.7 vs. 23.7 ± 2.3 kg/m^2^, *p* = 0.04, E2 decrease vs. E2 increase, respectively). Since high BMI is associated with low T, it is likely that the lower levels of T in individuals with high BMI will be substrate‐limiting and result in decreased or no change in estrogen expression with weight gain. Indeed, a strong correlation between T and E2 was observed in our study participants at baseline (*ρ* = 0.60, *p* = 0.002) which persisted after weight gain (*ρ* = 0.52, *p* = 0.008).

In obesity, low SHBG is implicated in increasing insulin resistance and cardiovascular risk and is suggested to be a marker of abdominal obesity (Brand et al., 2014; Gyawali et al., 2018; Moon et al., 2017; Perry et al., 2010; Wang et al., 2015). We demonstrate that modest weight gain is associated with decreases in SHBG. Our findings are, therefore, concordant with the increased cardiovascular risk associated with weight gain (Covassin et al., 2018; Romero‐Corral et al., 2010). These findings also align with a previous study of experimental weight gain which showed no changes in circulating T and estrogens and decreases in SHBG (Sato et al., 2014). However, changes in circulating SHBG were not associated with total or any regional fat depots in our study participants. We also show a dynamic inverse relationship between SHBG and leptin during weight gain. These findings are consistent with prior cross‐sectional studies showing an inverse relationship between leptin and SHBG (Abdella & Mojiminiyi, 2017; Liu et al., 2017; Vanbillemont et al., 2012), and suggest a causal relationship. Nevertheless, further studies are needed to support any direct role of leptin in regulating SHBG expression.

A consequence of SHBG lowering may be increased bioavailability of E2 and T. We show that modest weight gain is associated with increases in E2/SHBG and T/SHBG ratios. These findings seem contradictory to the observations from the experimental study examining the differential effects of T and estrogens in healthy males, which demonstrate that estrogen deficiency contributes to increased adiposity (Chao et al., 2016; Finkelstein et al., 2013). Finkelstein et al. (2013) observed that fat accumulation begins with mild gonadal deficiency but is primarily related to decreases in E2 levels. Our study shows increases in bioavailable T and E2 with modest increases in body weight and fat mass in healthy males. These increases in bioavailable gonadal hormones in early stages of weight gain may be an early adaptive response aimed at limiting increases in body weight and detrimental effects of weight gain in conditions of surplus calories. Evidence from animal studies suggest that estrogens play an important role in regulating food intake and energy expenditure, thereby regulating body weight (Rubinow, 2017). Specifically, increases in estrogens can decrease food intake via modulating central leptin and insulin sensitivity as well as directly contribute to hypophagia (Clegg et al., 2006; Gao et al., 2007; Rubinow, 2017). Estrogens have also been shown to increase voluntary activity and resting energy expenditure in male mice (Cederroth et al., 2007; Ogawa et al., 2003; Rubinow, 2017). Nevertheless, consistent with findings from Finkelstein et al. (2013) showing E2 deficiency leads to increased adiposity, participants with low baseline E2/SHBG showed higher increases in upper‐body fat mass during overfeeding‐induced weight gain. Conversely, participants with higher E2/SHBG at baseline showed lesser increase in upper‐body fat mass during weight gain. This suggests protective effects of E2 on upper‐body fat mass gain during excess calorie consumption.

Recent studies have shown that SHBG also functions as a hormone and is capable of inducing cellular signaling pathways independent of its interaction with sex hormones (Qu & Donnelly, 2020). Notably, overexpression of SHBG in an animal model is shown to protect against high‐fat diet‐induced weight gain and alterations in glucose metabolism (Saez‐Lopez et al., 2020). SHBG is also demonstrated to have direct lipolytic and anti‐inflammatory action in adipose tissue and macrophages (Saez‐Lopez et al., 2020; Yamazaki et al., 2018). Considering the role of SHBG in lipolysis, it is likely that the suppression of SHBG during early stages of weight gain may be an adaptive mechanism to ensure safe storage of excess energy in adipose tissue. However, in the long term this adaptation may not be beneficial as it may contribute to inflammation and accumulation of lipid in non‐adipose tissue such as liver as well. Indeed, increasing SHBG is being considered as a therapeutic strategy for the treatment of non‐alcohol fatty liver disease (Simo et al., 2015).

The strength of our study is in the longitudinal design, which allowed examination of the cross‐sectional as well as dynamic relationships between changes in regional fat depots with gonadal hormones. The inclusion of only healthy males in the study is also critical as it allows identification of early adaptive responses to calorie excess. Our study has several limitations including the relatively modest sample size and measurement of only circulating levels of T, E2, and E1. While the original study was designed to examine effects of weight gain on cardiovascular risk, we utilized the existing samples to longitudinally examine the effects of weight gain on sex hormones to contribute to existing knowledge related to sex hormones and body composition and provide a foundation for future studies that may use hormone supplementation to reduce abdominal adiposity. Considering the exploratory nature of the study, we did not measure localized changes in adipose tissue, nor did we measure molecular and cellular changes in adipose tissue mediated by weight gain, which may have provided mechanistic insights into estrogen‐mediated changes in adipose tissue metabolism during weight gain.

To conclude, our study highlights the complex interplay between body composition, T, E2, SHBG, and leptin. An in‐depth understanding would be critical to develop individualized treatment strategies relevant to reducing cardiovascular risk in males with obesity with or without hypogonadism. Clinically, the use of aromatizable androgens would be preferable only if the patient has low T and low E2, but not if the patient has low T in the presence of high E2.

## DISCLOSURES

Dr. Sert‐Kuniyoshi is a full‐time employee of Philips Respironics. All other authors have no conflict of interest to declare.

## AUTHORS’ CONTRIBUTIONS

Prachi Singh, Naima Covassin, Ravinder J. Singh, Michael D. Jensen, and Virend K. Somers designed the study; Fatima H. Sert‐Kuniyoshi, Abel Romero‐Corral, and Diane E. Davison conducted the research; Prachi Singh analyzed the data; Prachi Singh, Naima Covassin, Kara L. Marlatt, and Virend K. Somers wrote the paper; Prachi Singh had primary responsibility for the final content. All authors read and approved the final manuscript.
